# Misperception of maternal COVID-19 test status as a barrier to recruitment for an observational cohort study of mother–preterm infant dyads

**DOI:** 10.1017/cts.2024.572

**Published:** 2024-10-08

**Authors:** Anne L. Smazal, Alison E. Almgren-Bell, Jonah M. Silverglade, Lauren B. Bonner, Ann Dozier, Linda Van Horn, Jami Josefson, Daniel T. Robinson

**Affiliations:** 1 Department of Pediatrics, Northwestern University Feinberg School of Medicine, Chicago, IL, USA; 2 Ann & Robert H. Lurie Children’s Hospital of Chicago, Chicago, IL, USA; 3 Department of Preventive Medicine, Northwestern University Feinberg School of Medicine, Chicago, IL, USA; 4 University of Rochester, Rochester, NY, USA

**Keywords:** COVID-19, clinical research enrollment, clinical research participation, breastmilk, preterm infant

## Abstract

Enrollment into a prospective cohort study of mother–preterm infant dyads during the COVID-19 pandemic progressed slower than anticipated. Enrollment occurred during the first week after preterm birth, while infants were still hospitalized. We hypothesized that slower enrollment was attributable to mothers testing positive for COVID-19 as hospital policies restricted them from entering the neonatal intensive care unit, thus reducing interactions with research staff. However, only 4.5% of 245 screened mothers tested COVID-19 positive. Only 24.9% of those screened, far fewer than anticipated, were eligible for enrollment. Assumptions about pandemic-related enrollment barriers were not substantiated in this pediatric cohort.

## Introduction

Clinical investigations enrolling mother–infant dyads, particularly those focused on preterm infant (PTI) outcomes, often complete the informed consent process during the PTI hospitalization in the neonatal intensive care unit (NICU). This includes approaching eligible families, obtaining informed consent, and completing enrollment procedures. Notably, workflows for inpatient pediatric units changed drastically in response to the COVID-19 pandemic. Beginning in April 2020 and for varying durations, many NICUs enforced strict family visitation rules even in the absence of positive tests for SARS-CoV-2, the virus that causes COVID-19. This reduced time parents spent in the NICU, subsequently disrupting contact between research staff and parents [1–3]. More lasting policies for many NICUs restricted visitation by mothers testing positive [3].

A prospective cohort study of mother–PTI dyads provides opportunity to assess pandemic-related influences on enrollment into pediatric clinical research during COVID-19. During monthly meetings for such a study, investigators identified lower than anticipated enrollment rates and considered a multifactorial effect of the pandemic. We proposed that one measurable impact resulted from NICU policies restricting parental visitation during the enrollment window, specifically those with a positive test for SARS-CoV-2. We hypothesized that test positivity was common among those screened, irrespective of eligibility. Therefore, to evaluate this potential influence of the COVID-19 pandemic on pediatric research participation, we aimed to (1) measure rates of positive tests for SARS-CoV-2 among screened mothers of PTI hospitalized in a NICU and (2) explore whether dyads were deemed ineligible for enrollment more frequently than anticipated.

## Materials and methods

### Study population and design

This study reports prospectively collected data from the first 8 months of screening, late October 2021–June 2022, for an ongoing prospective cohort study enrolling mother–singleton PTI dyads at an urban, tertiary care delivery hospital in Chicago, Illinois. This screening period was chosen for study given consistency in hospital policies through June 2022, after which policies began to change. The study’s primary aims focus on breastmilk composition and measures of PTI adiposity related to maternal prepregnancy body mass index (BMI). Screening procedures facilitate enrollment of dyads in to one of two groups, a primary group in which delivery occurs at 28^0/7^−31^6/7^ weeks of gestation (goal *n* = 42 dyads) and a reference group in which delivery occurs at 34^0/7^−36^6/7^ weeks of gestation (goal *n* = 30 dyads). During the study, mothers provide expressed breastmilk across the first lactation month. Initial samples are obtained while mothers visit in the NICU. Final milk samples are obtained in the NICU or via courier home pickup depending on duration of infant hospitalization. Infants undergo standardized anthropometric measurements until NICU discharge. Local Institutional Review Boards approved this study (IRB#2021-4309; STU00214416) prior to participant enrollment. IRB approval included waiver of informed consent to collect deidentified information of all dyads screened, including those ineligible or who declined to participate, for purposes of study progress reports of demographics and clinical characteristics and comparison to those expected at study design.

### Screening/recruitment procedures and relevant hospital policies during the study period

**Figure 1.**
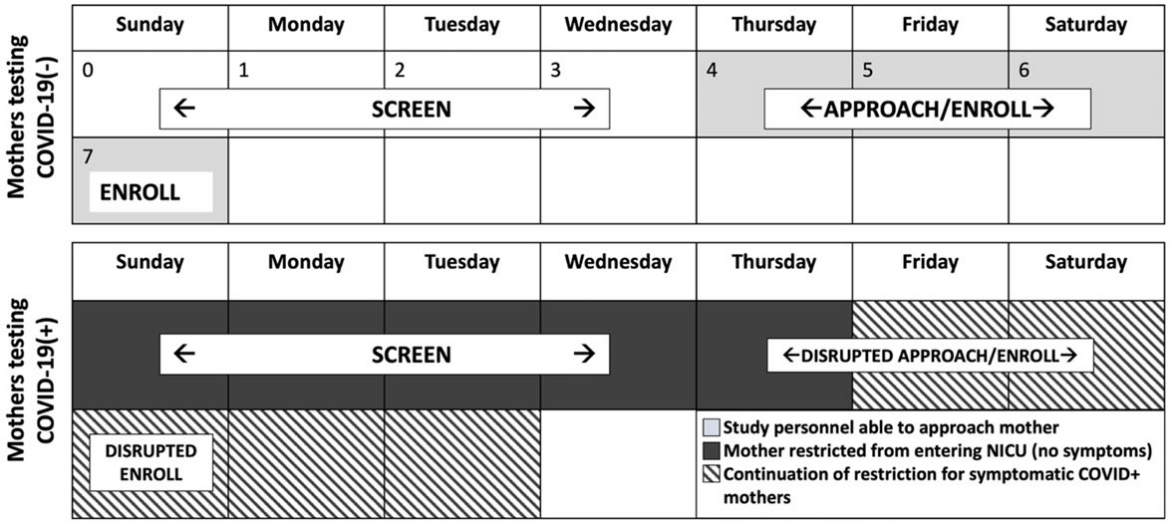
Timeline of study enrollment and neonatal intensive care unit visitor restrictions in place during screening based on COVID-19 test result. Study screening procedures begin on infant day 0 which is the infant’s day of birth. COVID-19(-) indicates a negative test result for SARS-CoV-2; COVID-19(+) indicates a positive test result. Mothers who test negative for COVID-19 can be approached beginning four days after delivery and ideally are enrolled by infant day 7. The example of mothers testing COVID-19(+) shows the scenario of a positive test result on the day of delivery. Mothers testing positive for COVID-19 are restricted from entering the NICU for at least 5 days after the positive test result, longer if symptomatic, limiting in-person interaction with study personnel and disrupting enrollment which ideally occurs by day 7. Those with symptoms are restricted for a minimum 10 days (as shown with hatched boxes) or until complete symptom resolution. NICU = neonatal intensive care unit.

During the screening period through June 2022, hospital policies required testing of all women for SARS-CoV-2 (subsequently referred to as COVID-19) by nasopharyngeal sampling and polymerase chain reaction assay upon admission for delivery. NICU policies restricted parent visitation for a minimum of 5 and up to 10 days after a positive test result (Figure 1). Beginning with study initiation in October 2021, electronic medical records (EMR) of all infants delivered at 28^0/7^−31^6/7^ or 34^0/7^−36^6/7^ weeks of gestation and their mothers were screened to assess eligibility. Screening of dyads occurred during lactation initiation, between the infant’s day of birth (day 0) and continuing during the next 96 hours. Then, study staff approached eligible parents with the goal of obtaining informed consent by infant postnatal day 7. Mothers unable to visit the NICU because of the 5-day minimum restriction, or longer with symptoms, when testing positive for COVID-19 were contacted by phone as an initial approach about the research opportunity. Eligible mothers were age ≥18 years and delivered after a singleton pregnancy. Exclusion criteria included prepregnancy BMI<18.5 kg/m^2^, diagnosis of gestational diabetes mellitus (GDM), and decision not to provide breastmilk as the primary form of nutrition for the infant. Mothers testing positive for COVID-19 were not precluded from enrolling or ongoing participation. Eligible infants required a birthweight for gestational age between the 10^th^ and 90^th^ percentiles on sex-specific growth curves [4], no respiratory support or infection (reference group only), no aneuploidy, and low likelihood of mortality by the medical team’s assessment. Documentation of reason(s) for ineligibility specified whether related to the parent or infant. Based on a previous cohort study of mother–PTI conducted at our center with somewhat similar eligibility criteria yet completed prior to the pandemic, we anticipated 88% of those screened would be eligible for enrollment [5].

### Outcomes

Our primary outcome for this brief report was rate of positive COVID-19 test among those screened for study eligibility. Measured outcomes included selected demographic and clinical variables from the EMR, COVID-19 test result upon hospital admission for delivery, and COVID-19 vaccination status. Any dose(s) qualified as having received a COVID-19 vaccine. The secondary study outcome was reason(s) for dyad ineligibility. Additional variables collected included maternal age at delivery, race and ethnicity (obtained from the EMR for those not enrolled or by self-report for those enrolled), gravidity and parity, prepregnancy BMI, delivery mode, use of assisted reproductive technology, hypertensive disorders of pregnancy (chronic hypertension preceding pregnancy, gestational hypertension, and preeclampsia). Prepregnancy weight was defined as a weight obtained within 60 days prior to conception (preferred) or between 0 and 11 weeks of gestation as recorded in the EMR (11), or self-report if not specified in the EMR. Prepregnancy BMI was classified as: normal, ≥ 18.5 and < 25 kg/m^2^; overweight, ≥ 25.0 and < 30.0 kg/m^2^; and obese, ≥ 30.0 kg/m^2^) [6].

### Statistical analysis

Descriptive statistics summarized demographic and clinical variables of interest by eligibility and enrollment status. Distributions of continuous variables were compared across three dyad groups (ineligible; eligible and declined; eligible and enrolled) using analysis of variance (ANOVA) or Kruskal–Wallis tests, for non-normally distributed variables. Chi-squared tests or Fisher’s exact tests, as appropriate, compared proportions of categorical variables across groups. As recording of variables for those not enrolled relied on data exclusively in the EMR, missing data occurred; statistical comparisons utilized available data only. Descriptive statistics also summarized reasons for ineligibility and characteristics of the subgroup testing positive for COVID-19. Two sample tests of proportions compared eligibility rates and enrollment rates between our study and a comparable cohort study conducted prior to the pandemic [5]. All analyses assumed a two-sided type I error rate of 0.05. As this was secondary analysis of an ongoing cohort study, no sample size calculations were performed.

## Results

### COVID-19 positive test status

**Figure 2.**
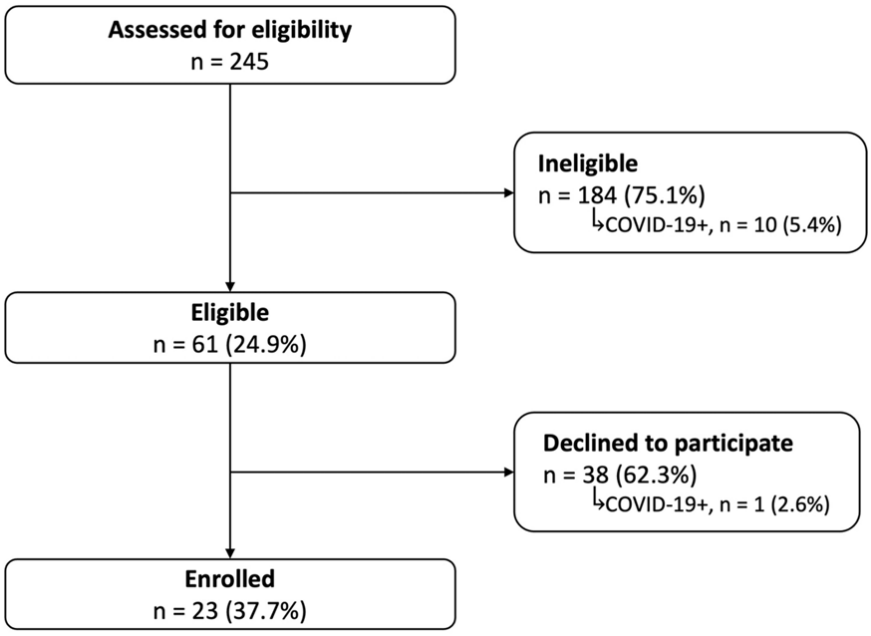
Flow diagram indicating the number of dyads assessed for study eligibility, their final determination as well as maternal COVID-19 positive tests during the 8-month screening period.

During the screening and recruitment period of October 2021–June 2022, 245 mother–PTI dyads were screened for eligibility (Figure 2) and approximately one-quarter met initial enrollment criteria. Of those screened, 11 mothers (4.5%) tested positive for COVID-19 on admission for delivery. Ten of those with a positive test were categorized as ineligible for enrollment; 4 of the 10 were ineligible for enrollment due to inability to consent within the desired enrollment window. One eligible mother with a positive test declined to enroll. Notably, the majority of the screened population had prior COVID-19 vaccines (77%) as did most of the 11 mothers testing positive for COVID-19 (73%).

### Eligibility and enrollment

Of the 61 eligible dyads, 23 (37.7%) enrolled in the study. Table 1 compares maternal characteristics between ineligible dyads, eligible dyads who declined participation, and eligible dyads who enrolled. Though not exclusion criteria, hypertensive disorders of pregnancy were common among those eligible with 49.2% experiencing any of chronic or gestational hypertension or preeclampsia. Most screened dyads were ineligible (Table 2) primarily due to maternal factors, including GDM, minimal or no breastmilk availability, or multiple gestation pregnancies. Small for gestational age (birthweight < 10^th^ percentile) infant status occurred as expected based on distributions on a growth curve. The proportion of screened mothers who were eligible was significantly lower in our study compared to a comparable cohort study of PTIs implemented at our center prior to the pandemic (Prior cohort: 88%; Current cohort: 24.9%, *p* < 0.001). The proportion enrolled of those eligible in the prior and this cohort was not significantly different (Prior cohort: 43%; Current cohort: vs. 37.7%, *p* = 0.795). All enrolled in the current study completed mandatory study procedures.

**Table 1.** Maternal demographic and health characteristics by enrollment status

	Eligible *n* = 61		Not eligible *n* = 184	*p-V*alue
	Declined *n* = 38	Enrolled *n* = 23		
**Study group**				
Primary, *n* (%)	11 (28.9)	7 (30.4)	34 (18.5)	0.187
Reference, *n* (%)	27 (71.1)	16 (69.6)	150 (81.5)	
**Maternal age, mean (SD)^1^**	33.65 (5.17)	32.36 (4.56)	33.90 (5.63)	0.446
**Race, *n* (%)^2^**				0.270
Asian	3 (7.9)	0 (0.0)	18 (10.4)	
Black	7 (18.4)	0 (0.0)	35 (20.2)	
Native American/Alaskan Native	0 (0.0)	0 (0.0)	1 (0.6)	
Other/Multiple	7 (18.4)	6 (27.3)	36 (20.8)	
White	19 (50.0)	16 (72.2)	83 (48.0)	
**Ethnicity, *n* (%)^3^**				0.525
Hispanic/Latino	6 (16.7)	6 (27.3)	43 (25.0)	
Not Hispanic/Latino	30 (83.3)	16 (72.7)	129 (75.0)	
**Primigravid, *n* (%)**	12 (31.6)	8 (34.8)	62 (33.9)	0.956
**Prepregnancy BMI, median [IQR]^3^**	25.46 [23.29, 26.58]	25.33 [23.03, 26.44]	26.48 [22.56, 33.16]	0.411
**Prepregnancy BMI group^4^**				0.296
Underweight	0 (0.0)	0 (0.0)	5 (3.5)	
Normal	11 (44.0)	9 (40.9)	50 (35.0)	
Overweight	9 (36.0)	9 (40.9)	37 (25.9)	
Obese	5 (20)	4 (18.2)	51 (35.7)	
**Delivery mode, *n* (%)^5^**				0.803
Vaginal	18 (48.6)	11 (47.8)	76 (43.4)	
Cesarean	19 (51.4)	12 (52.2)	99 (56.6)	
**Assisted reproductive technology, *n* (%)**	6 (15.8)	1 (5.3)	24 (13.2)	0.529
**Chronic hypertension, *n* (%)^6^**				0.058
No	35 (92.1)	23 (100.0)	152 (84.0)	
Yes	3 (7.9)	0 (0.0)	29 (16.0)	
**Gestational hypertension, *n* (%)^7^**				0.007
No	35 (92.1)	22 (95.7)	136 (74.7)	
Yes	3 (7.9)	1 (4.3)	46 (25.3)	
**Preeclampsia, *n* (%)^6^**				0.850
No	22 (57.9)	15 (65.2)	109 (60.2)	
Yes	16 (42.1)	8 (34.8)	72 (39.8)	
**Tested positive for COVID-19**	1 (2.6)	0 (0.0)	10 (5.4)	0.413
**Received any vaccine for COVID-19^8^**				0.339
No	1 (16.7)	0 (0.0)	36 (25.0)	
Yes	5 (83.3)	6 (100.0)	108 (75.0)	

Percentages reflect proportion of non-missing data.

BMI = body mass index.

1 Missing values: declined *n* = 1; not eligible *n* = 1.

2 Missing values: declined *n* = 2; enrolled *n* = 1; not eligible *n* = 11.

3 Missing values: declined *n* = 2; enrolled *n* = 1; not eligible *n* = 12.

4 Missing values: declined *n* = 13; enrolled *n* = 1; not eligible *n* = 41.

5 Missing values: declined *n* = 11; not eligible *n* = 9.

6 Missing values: not eligible *n* = 3.

7 Missing values: not eligible *n* = 2.

8 Missing values: declined *n* = 32; enrolled *n* = 17; not eligible *n* = 40.

**Table 2.** Exclusion criteria for mother–preterm infant dyads deemed not eligible during screening

Exclusion criterion	Not eligible *n* = 184
**Maternal criteria**	
Multiple gestation	33 (17.9)
Gestational diabetes mellitus	39 (21.2)
Prepregnancy BMI<18.5	1 (0.5)
Delivery outside of center	1 (0.5)
Age<18 years	1 (0.5)
No breastmilk	18 (9.8)
Minimal breastmilk	15 (8.2)
Low milk status: minimal or none	33 (17.9)
Out of enrollment window	25 (13.6)
**Infant criteria**	
Birthweight<10^th^ percentile for gestational age	25 (13.6)
Birthweight>90^th^ percentile for gestational age	16 (8.7)
Birthweight percentile either<10^th^ or>90^th^	41 (22.3)
High likelihood of mortality	2 (1.1)
Respiratory support*	15 (8.2)
Infant infection*	3 (1.6)
Known aneuploidy	1 (0.5)
Other	1 (0.5)

Values are *n* (%) of total and each indication is not mutually exclusive.

BMI = body mass index.

* Relevant to screening for reference group only.

## Discussion

With expectations that high rates of positive COVID-19 tests were influencing enrollment, in fact, positive tests among those screened appeared to have little to no bearing on enrollment in a prospective cohort study of mother–PTI dyads initiated during the COVID-19 pandemic. As studied at the same center in which our cohort study occurs, vaccination rates among pregnant women were over 75% [7], some of the highest rates nationally. We speculate this high vaccination rate may have influenced the low positive test rate among those screened. Even in consideration of parents who test negative, variation in pandemic-related policies imposed by hospitals across healthcare systems [2,3] may have variably influenced workflows and enrollment for perinatal clinical research implemented in healthcare settings outside of our center.

While test positivity was not a factor in this study, which reduces concern of disruptions to approaching eligible dyads, other implications of COVID-19 may have contributed to unexpectedly low enrollment. Specifically, fewer than anticipated eligible participants were observed. Higher eligibility occurred during a comparable observational cohort study of PTIs conducted at our center [5]. This discrepancy may be partially due to our projections, grant development, and submission which occurred prior to the pandemic.

The eligibility rate of those screened was distinctly low, primarily due to maternal characteristics, prompting some consideration of changing health characteristics related to the pandemic. Several cohorts report increased rates of GDM during the pandemic [8–11]. Our anticipated rates were 10%; cohorts during the pandemic report rates as high as 14.5% [8–10]. During screening, rates of GDM at our center were, without explanation, considerably higher than observed in the past. Poor fetal growth in the USA also occurred more frequently during the pandemic [11], possibly secondary to increased incidence of hypertensive disorders of pregnancy during local stay-at-home directives [11–13]. Our cohort similarly noted a high proportion of both gestational hypertension and preeclampsia. Collectively these findings suggest pandemic-related lifestyle alterations and stress [14,15] may have contributed to adverse metabolic outcomes in pregnancy, thereby influencing numbers eligible. Also, patterns of healthcare utilization by pregnant populations during the pandemic included reduced inpatient admissions [16,17] which may have impacted potential participant availability.

Limitations include small sample size and selection bias inherent to recruitment from an urban, tertiary care referral center for high-risk pregnancies. As mentioned, we were limited in capturing other effects from COVID-19 on enrollment. Namely, potential stress and health implications that may have influenced eligibility remain unmeasured. Informal feedback from parents not enrolling included sentiments of “feeling overwhelmed” as the reason for declining to participate. This does not clarify whether circumstances of preterm birth versus the pandemic, or both, contributed to these feelings. More systematic methods for collecting reasons for not participating may better define contributing factors to these overwhelmed feelings.

In conclusion, the slower than anticipated pace of enrollment in a cohort study of mother–PTI dyads cannot be attributed specifically to positive tests for COVID-19 causing disruptions to contact with parents of infants hospitalized in our NICU. These findings highlight needed stepwise assessments of recruitment processes to identify and address potential barriers when extraordinary circumstances are believed to impact research progress. Many investigators have commented that enrollment patterns differ before and since COVID-19. Given our findings, there may be misperceptions about contributing factors. Enrollment differences may be due to factors entirely unrelated to the pandemic [18]. Publication by additional studies recruiting during the pandemic will confirm or refute our conclusions. We anticipate that such collective reporting by similarly affected studies will improve objective definitions of challenges faced by pediatric clinical research teams and improve screening and enrollment processes, especially for studies focused on families affected by preterm birth.
